# Treatment outcome and associated factors of bacterial meningitis at pediatric wards of southwestern Ethiopian hospital: a prospective observational study

**DOI:** 10.1186/s40780-021-00224-9

**Published:** 2021-11-15

**Authors:** Firomsa Bekele, Anuwar Ahmed, Abas Kedir, Tadesse Sheleme

**Affiliations:** 1 Department of Pharmacy, College of Health Science, Mettu University, Mettu, Ethiopia; 2 Dedo Primary Hospital, Oromia, Ethiopia; 3 Ginir Primary Hospital, Oromia, Ethiopia

**Keywords:** Associated factors, Bedele general hospital, Meningitis, Pediatric ward, Treatment outcome, Ethiopia

## Abstract

**Background:**

Meningitis is a common infectious cause of morbidity and mortality in pediatric age-groups. Acute bacterial meningitis is considered a medical emergency, because it is a life-threatening infection that requires immediate treatment. Therefore the study was aimed to assess the magnitude and predictors of poor treatment outcome among pediatric patients admitted to Bedele General Hospital.

**Methods:**

A prospective observational study was conducted at pediatric wards of Bedele General Hospital from February 12, 2020 to August 11, 2020. Lumbar puncture, in the absence of contraindications, was performed under aseptic conditions for all patients with suspected bacterial meningitis to collect cerebrospinal fluid specimen. Multivariable logistic regression was used to determine the predictors of poor treatment outcome.

**Result:**

Of the 196 pediatric patients involved, 112(57.1%) were male and the mean and standard deviation of their age was 6.09 ± 4.46. Regarding to their clinical profile, a total of 101(51.5%) were completely immunized and 115(58.7%) were given corticosteroid during their treatment. In our study the most frequently occurred clinical manifestation of meningitis was fever 164(83.67%), neck rigidity149 (76.02%), and irritability 122(62.24%). Regarding to their pharmacotherapy, the most commonly prescribed antibiotics were Ampicillin 104(24.82%), and Gentamycin 102(24.34%). The magnitude of good treatment outcome was 132(67.35%) whereas 64(32.65%) were poorly controlled. The presence of comorbidity (AOR = 3.64, 95CI%:1.83–7.23,P = < 0.001),corticosteroid use (AOR = 2.37, 95CI%:1.17–4.81,*P* = 0.017) and oxygen administration (AOR = 3.12, 95CI%: 1.34–7.25, *P* = 0.008) was a predictor of meningitis treatment outcome.

**Conclusion:**

The treatment outcome of meningitis was good in of two-third of the patients. It was found that the presence of comorbidity, the administration of oxygen and use of corticosteroid was predictors of the treatment outcomes of bacterial meningitis in children. Therefore, in patients with these factors, appropriate meningitis treatment should be encouraged and locally applicable treatment guidelines should be prepared to improve patient outcome. Finally, the meningitis patients should be given corticosteroid and oxygen as treatment and special attention should be given for patients having co-morbidities.

## Background

Meningitis is one of the most common types of central nervous system (CNS) infection and it is an inflammation of the meninges; that involves the subarachnoid space or spinal fluid [1, 2]. Definitive diagnosis of acute bacterial meningitis (ABM) is often dependent on cerebrospinal fluid (CSF) detail report and its culture by performing lumbar puncture [3].

The region in Sub- Saharan Africa, including Ethiopia, is known as the “meningitis belt” because of the high prevalence of meningitis in the area [2, 4]. More than one million cases of acute bacterial meningitis (ABM) amongst adults and children occur annually in sub-Saharan Africa (SSA) [5].

Despite the availability of newer potent antibiotics and preventive strategies, the morbidity and mortality and complications secondary to ABM continued to rise [1, 6]. It is a common infectious cause of morbidity and mortality in pediatric age-groups [7]. Each year, it affects about 2.81 million children, especially < 5 years of age [2]. Meningitis is one of the top ten causes of death among Ethiopian infants [8].

Bacterial meningitis is one of the most potentially serious infections occurring in infants and children [1]. About one third of meningitis patients developed complications like hearing impairment, seizure disorders, developmental delays and learning and behavioral problems [2, 9].

Different factors were associated with poor outcomes of meningitis patients like the duration of disease, age, immune status of the patient, timing of antibiotic initiation, type of microorganism, rapid diagnosis and an early treatment [10, 11].

Even though the diagnosis of the disease is a difficult task in pediatrics, it is considered a medical emergency [3, 9]. Any delay in the initiation of treatment could be fatal and in most resource limited settings the mortality of untreated bacterial meningitis approaches 100% [1, 4]. Therefore, early diagnosis, identification of the pathogen, and time to initiation of adequate antibiotic therapy are important variables that can improve the clinical outcomes of bacterial meningitis in children [4, 9].

Despite the conjugate vaccine against *Haemophilus influenzae serotype b, Streptococcus pneumoniae* (pneumococcal conjugate vaccine (PCV),were introduced in Ethiopia to prevent bacterial meningitis, most patients treated as bacterial meningitis did not receive a proper diagnostic workup and the choice of antibiotic was blindly without identifying the specific strains of causative agents that may result in poor treatment outcomes and lead to antibiotic resistance [12]. Therefore, the aim of this study was to identify the magnitude and associated factors of poor treatment outcomes among children admitted with meningitis to Bedele General Hospital (BGH), Ethiopia.

## Methods

### Study area, design and period

A prospective observational study was conducted at Bedele General hospital (BGH) from February 12, 2020 to August 11, 2020. BGH is found in Bedele town, South West Oromia, Ethiopia which is found at 426 km from Addis Ababa. There are different wards and clinics within BGH; those include internal medicine ward, surgery ward, pediatric ward, gynecology and obstetrics ward, Ante natal clinic, dental clinics, tuberculosis clinic, anti-retro viral therapy clinic and ophthalmic clinic.

### Study participants and eligibility criteria

Patients whose age was less than or equal to 14 years and complete registration charts who were diagnosed bacteriologically by physicians as having of BM and treated for BM during the study period were included whereas patients whose back ground information was incomplete or no drug orders on their charts, pediatrics with treatment outcome not stated, pediatrics with TB meningitis and patients who completed treatment for possible bacterial meningitis but did not have supportive laboratory or clinical evidences were excluded. The diagnosis of bacterial meningitis was confirmed by performing latex agglutination to detect the presence of bacterial antigens in the CSF.

The latex agglutination test was done using the bacterial antigen kit (Wellcogen1 Bacterial antigen kit, UK) designed to detect 5 groups of bacteria: *Group B Streptococcus (GBS), Haemophilus influenza type B (Hib), Streptococcus pneumoniae, Neisseria meningitidis ACYWand Neisseria meningitides B/Escherichia coli* K1.

### Study variables and outcome endpoints

Meningitis treatment outcome was a primary outcome. The patients were daily followed to assess the disappearances of meningitis sign and symptoms. Besides this, the patient was followed by a neurosign chart that includes vital signs, bulging fontanelle, photophobia, a positive Kernig’s or Brudzinski’s sign, seizure, headache, and nuchal rigidity. The outcome of the patients and the presence of the complications among pediatrics were evaluated during their hospital stay [2].

### Sample size and sampling technique

Single population proportion formula was used to calculate the required sample size by considering the following assumptions: Proportion of meningitis treatment outcome *P* = 0.77 [2], 95% confidence level, and 5% margin of error (absolute level of precision).

$$ {\displaystyle \begin{array}{l}\mathrm{n}=\frac{\left(\mathrm{Za}/2\right)2\mathrm{p}\left(1\hbox{-} \mathrm{p}\right)}{\mathrm{d}2}\ \\ {}\mathrm{z}=1.96\\ {}\mathrm{P}=77\%(0.77)\\ {}\mathrm{d}=0.05\\ {}\mathrm{n}=\frac{(1.96)2(0.77)(0.23)}{(0.05)2}=272\end{array}} $$

Where;

n = Sample size

P = Proportion of meningitis treatment outcome = 77%

Z = Z is standardized normal distribution value at the 95% CI: 1.96

d = The margin of sample error tolerated = 5%

The expected number of populations in the study period (N), based on the average number of patients coming to the hospital in 6 months was 520. The corrected sample size (nf), was calculated by using correction formula as follows:

$$ \mathrm{nf}=\frac{\left(\mathrm{n}\ast \mathrm{N}\right)}{\left(\mathrm{n}+\mathrm{N}\right)} $$

$$ \mathrm{nf}=\frac{\left(272\ast 520\right)}{\left(272+520\right)} $$

nf = 178.58. After adding a 10% contingency it becomes 196. A consecutive sampling technique was used to include study participants.

### Data collection process and management

A semi-structured data collection tool was prepared to collect the data. Three medical doctor and two clinical pharmacists were recruited for data collection; one clinical pharmacist was assigned to supervise the data collection process. Lumbar puncture, in the absence of contraindications, was performed under aseptic conditions for all patients with suspected ABM to collect CSF specimen. To assure the consistency of the data collection tool it was pretested at nearby hospital called Mettu Karl Referral Hospital prior to normal data collection.

### Data processing and analysis

The data were entered into a computer using EPI-data version 3.1. The principal investigators had daily checked and clean the data. The data was then exported to statistical software for social sciences (SPSS) 24.0 for analysis. Multivariable logistic regression was used to analyze the variable by using crude odds ratio (COR) and adjusted odds ratio (AOR) with 95% CI. All variables associated with the meningitis treatment outcome at a *P*-value ≤0.25 on the bivariate analysis were entered into a multivariable logistic regression analysis to control for confounders. Finally, the predictors of meningitis treatment outcome were declared if a *P* value of ≤0.05.

### Operational definitions

- ❖ **Pediatrics:** According to the current study, included infants and children aged from 1 day to 14 years [1, 2].
- ❖ **Good outcome:** Which means improvement without acute complications [1, 2].
- ❖ **Poor outcome:**Death within the ward, developed acute neurologic complications during treatment or at discharge, self-discharge before completion of treatment, referred to higher facility for further management, left against medical advice without adequate improvement and prolonged hospitalization [1, 2].

## Results

### Socio-demographic factors and clinical characteristics of study population

A total of 196 meningitis patients were admitted to pediatric ward. Of these, 112(57.1%) were male and 84(42.9%) were females and the mean and standard deviation of their age was 6.09 ± 4.46. A total of 102(52%) were from urban area whereas 94(48%) were from rural. Regarding to their clinical profile, a total of 101(51.5%) were completely immunized and 115(58.7%) were given corticosteroid to treat bacterial meningitis (Table 1).

**Table 1:** Socio-demographic factors and clinical characteristics of meningitis patients admitted to pediatric ward of BGH from February 12, 2020 to August 11, 2020

Variables		Frequency	Percentage
Age	< 2 months	16	8.2
	> = 2moths	180	91.8
Sex	Male	112	57.1
	Female	84	42.9
Residence	Urban	102	52
	Rural	94	48
Duration of illness before admission	< 72 h	92	46.9
	> = 72 h	104	53.1
Immunization history	Complete	101	51.5
	Incomplete	95	48.5
Co-morbidity	No	70	42.33
	Yes	126	35.7
Corticosteroid used for meningitis treatment	No	81	41.3
	Yes	115	58.7
Oxygen administration	No	162	82.7
	Yes	34	17.3
Pneumonia	Yes	67	34.18
	No	129	65.82
Sepsis	Yes	107	54.59
	No	89	45.41
Sever acute malnutrition	Yes	33	16.84
	No	163	83.16
Malaria	Yes	36	18.37
	No	160	81.63
Typhoid fever	Yes	45	22.96
	No	151	77.04
Cellulitis	Yes	62	31.63
	No	134	68.37

Among those who had their CSF analyzed (102), causative bacteria were detected in 67 (65.69%) patients. *Group B Streptococcus* 18 (26.87%) and *Haemophilus influenzae type B* 17 (25.37%) were commonly identified whereas *Listeria monocytogenes* 8(11.94%) was infrequently isolated (Table 2). Regarding to the efficacy of the bacteriology, *Group B streptococci*(33.33%) and *Listeria monocytogenes 3(37.50%)*were more detected in newborns. *Haemophilus influenzae type B* 7(41.18%) and *Escherichia coli 6(54.55%)* were predominant in infants whereas *Neisseria meningitides* was common among children age between 1 and 11 years that accounts 7(53.85%).

**Table 2:** The commonly occurred bacterial species of meningitis patients

Bacterial aetiology	Frequency	Percentage
*Group B Streptococcus*	18	26.87
*Haemophilus influenzae type B*	17	25.37
*Neisseria meningitidis*	13	19.40
*Escherichia coli*	11	16.42
*Listeria monocytogenes*	8	11.94

In our study, the most frequently occurred clinical manifestation of meningitis was fever 164(83.67%), neck rigidity149 (76.02%), and irritability 122(62.24%) whereas bulging fontanel was the rare clinical presentation of meningitis patients (Table 3).

**Table 3:** The commonly occurred clinical presentation among meningitis patients admitted to pediatric ward of BGH from February 12, 2020 to August 11, 2020

Variables		Frequency	Percentage
Fever	Yes	164	83.67
	No	32	16.33
Vomiting	Yes	105	53.57
	No	91	46.43
Poor feeding	Yes	102	52.04
	No	94	47.96
Neck rigidity	Yes	149	76.02
	No	47	23.98
Seizure	Yes	45	22.96
	No	151	77.04
Irritability	Yes	122	62.24
	No	74	37.76
Bulged fontanel	Yes	7	3.57
	No	189	96.43

Regarding to their pharmacotherapy, the most commonly prescribed antibiotics were Ampicillin 104(24.82%), Gentamycin 102(24.34%) and Vancomycin75 (17.89%) whereas Cloxacillin 4(0.95%) was the least prescribed antibiotics for management of bacterial meningitis at Bedele General Hospital (Table 4). In our study, Ampicillin was changed to Metronidazole in three of the patients due to the drug allergy. Ampicillin provides good coverage for gram-positive cocci, including *group B streptococci (GBS), Listeria monocytogenes,* some strains of *Escherichia coli,* and *Haemophilus influenzae type B*. Gentamicin have good activity against gram negative bacteria like *Escherichia coli* whereas Cloxacillin is effective against gram positive bacteria like *Group B streptococci (GBS).* Third-generation Cephalosporins, such as ceftriaxone and cefotaxime are effective agents against gram-negative infections, *Escherichia coli,* and gram positive organisms, *Group B streptococci*. None of the Cephalosporins have any activity against *Listeria monocytogenes*; therefore, they should not be used alone for initial treatment. A combination of ampicillin and a third-generation cephalosporin is required. Vancomycin should be considered for *Group B streptococci*. The combination of Vancomycin and Ceftriaxone could provide adequate coverage for most penicillin-resistant pneumococci and beta-lactamase–resistant *Haemophilus influenzae type B.*

**Table 4:** The commonly prescribed antibiotics among meningitis patients admitted to pediatric ward of BGH from February 12, 2020 to August 11, 2020 (*N* = 419)

Antibiotics	Frequency	Percentage
Ceftriaxone	64	15.27
Ampicillin	104	24.82
Gentamycin	102	24.34
Amoxicillin	18	4.29
Azithromycin	9	2.15
Metronidazole	30	7.16
Cotrimoxazole	6	1.43
Cloxacillin	4	0.95
Vancomycin	75	17.89
Cefotaxime	7	1.67

### Determinants of meningitis treatment outcome among pediatric patients

In our study, the magnitude of good treatment outcome was 132(67.35%) whereas 64(32.65%) were poorly controlled. Pediatrics patients who had two or more disease and patients who didn’t given corticosteroid and oxygen to treat meningitis were more likely to had unfavorable treatment outcome. The patient having two or more disease was 3.64 more likely to had poor treatment outcome than their counterparts (AOR = 3.64, 95CI%:1.83–7.23,P = < 0.001). Pediatric population didn’t given corticosteroid had 2.37 more likely to experience poor treatment outcome than patients given corticosteroid (AOR = 2.37, 95CI%:1.17–4.81,*P* = 0.017). Similarly, patients didn’t administered oxygen had 3.12 more likely to had poor treatment control than their counterparts (AOR = 3.12, 95CI%: 1.34–7.25, *P* = 0.008) (Table 5).

**Table 5:** Bivariable and Multivariable logistic regression analysis result of factors associated with treatment outcomes of bacterial meningitis among pediatric patients admitted to pediatric ward of BGH from February 12, 2020 to August 11, 2020

Variables	Category	Treatment outcome		COR(95%CI)	AOR(95%CI)	*P*-value
		Good	Poor			
Age	< 2 months	11(8.33%)	5(7.81%)	1	0.44(0.12–1.63)	0.22
	> = 2 months	121(91.67%)	59(92.19%)	0.93(0.31–2.81)	1	
Sex	Male	73(55.30%)	39(60.94%)	0.79(0.43–1.46)	0.77(0.38–1.54)	0.46
	Female	59(44.70%)	25(39.06%)	1	1	
Residency	Urban	73(55.30%)	29(45.31%)	1	1	0.072
	Rural	59(44.70%)	35(54.69%)	0.67(0.37–1.22)	0.52(0.26–1.06)	
Duration of illness	< 72 h	58(43.94%)	34(53.13%)	1	1	0.066
	> = 72 h	74(56.06%)	30(46.87%)	1.45(0.79–2.63)	1.92(0.96–3.84)	
Oxygen use	Yes	15 (11.36%)	19(29.69%)	1	1	0.008
	No	117(88.64%)	45(70.31%)	3.29(1.54–7.04)	3.12(1.34–7.25)	
Immunization history	Complete	67(50.76%)	34(53.13%)	1	1	0.63
	Incomplete	65(49.24%)	30(46.87%)	1.10(0.61–1.99)	1.19(0.59–2.38)	
Corticosteroid used for meningitis treatment	Yes	69(52.27%)	46(71.86%)	1	1	0.017
	No	63(47.73%)	18(28.14%)	1.50(1.23–4.44)	2.37(1.17–4.81)	
Co-morbidity	No	97(73.48%)	29(45.31%)	1	1	< 0.001
	Yes	35(26.52%)	35(54.69%)	3.34(1.79–6.25)	3.64(1.83–7.23)	

*AOR* Adjusted Odd ratio; *CI* Confidence Interval; *COR* Crude Odd Ratio

## Discussion

Poor outcomes occur from meningitis in developing countries because of advanced disease at the time of presentation, co-existing malnutrition, antibiotic resistant bacteria, shortages of effective antibiotics and deficits in case management and staffing [7].

In our study, a total of 132(67.35%) meningitis patients had a good treatment outcome. This finding was lower than the study of Hiwot Fana Specialized University Hospital, Eastern Ethiopia,77% [2], Felege Hiwot Referral Hospital 85% [4] and Jimma University Specialized Hospital 71.7% [1]. The increased in poorly controlled meningitis in our study might be due to the empirical treatment of antibiotics and poor setting than aforementioned hospitals. This is due to the fact that above hospitals were a referral and specialized hospital that had adequate laboratory equipment’s to undergone bacterial culture before starting treatment and had sufficient health care workers than our study area, Bedele General Hospital.

In our study the most frequently occurred clinical manifestation of meningitis was fever 164(83.67%), neck rigidity149 (76.02%), and irritability 122(62.24%) whereas bulging fontanel was the rare clinical presentation of meningitis patients. This was similar with the study of Aga Khan University Hospital,Kenya, Hiwot Fana Specialized University Hospital, eastern Ethiopia and Felege Hiwot Referral Hospital [2–4]. This showed that the clinical presentation of meningitis looks similar despite different age classifications of pediatrics.

Antibiotics should be started immediately in meningitis since delay in the initiation of therapy introduces the potential for increased morbidity and mortality [13]. In BGH the most commonly used antibiotics was Ampicillin and Gentamicin which was similar with the study of Jimma University Specialized Hospital and Hiwot Fana Specialized University Hospital, eastern Ethiopia [2, 14]. This was might be due to the common causative pathogens across the setting and the preference of physicians to combine both antibiotics for synergisms which might result in poor treatment outcomes and lead to antibiotic resistance. During our study, in three of the patients Ampicillin was changed to Metronidazole due to the drug allergy.

Despite, the subsequent management of the patients should be guided by findings of CSF analysis; the antibiotics should be started early to reduce the unfavourable outcome. In our study, third-generation cephalosporins (cefotaxime or ceftriaxone) should be used more as empiric therapy of pediatric meningitis due to the coverage of *Neisseria meningitides, Streptococcus pneumonia,* and *Haemophilus influenza type B*, and penetrate CSF well. Ampicillin should be added in young infants less than 3 months old to cover *Listeria monocytogenes.*

Early prediction of an adverse outcome may help determine which children require more intensive or longer follow-up and may provide the physician with rationale for parental counseling about their child’s prognosis in an early phase of the disease [9].

The pediatric patients who had two or more disease had poor treatment outcome of their disease. This is inconsistent with the study of Jimma University Specialized Hospital and Hiwot Fana Specialized University Hospital, eastern Ethiopia [1, 2]. This is due to the possibility of drug-drug interactions as multiple drugs were prescribed to treat the co-morbidity which can affect the patient’s treatment outcome.

In our study patients who hadn’t used corticosteroid had a poor treatment outcome as compared to their counterparts. This finding was inconsistent with the study of Jimma University Hospital in southwest Ethiopia [15]. Similar study was obtained in Felege Hiwot Referral Hospital, northwest Ethiopia in which Children who did not receive corticosteroid medication were almost 8 times higher to develop poor outcome than those who received corticosteroid medication [4]. This was consistent with the recommendation of Infectious Diseases Society of America in which Dexamethasone therapy should be administered immediately after CSF is obtained [13]. This is due to the fact that corticosteroids are used as adjuvant treatment in childhood pyogenic meningitis to attenuate host inflammatory responses to bacterial invasion [16].

The patients who were didn’t administered oxygen had poor treatment outcome than patients given oxygen. This is inconsistent with the finding of Felege Hiwot Referral Hospital, northwest Ethiopia in which oxygen administration didn’t predict treatment outcome [4]. This is due to oxygen therapy can help patents with meningitis to heal by preventing hypoxia that decrease the risk of meningitis complication like shock and coma.

As the strength the study was a prospective which takes into account different laboratory investigations and neurosign chart to identify the treatment outcomes and as limitation the study was a single-center and its results cannot be generalized and the impact of drug therapy problems on treatment outcome was not identified.

## Conclusion

The treatment outcome of meningitis was good in of two-third of the patients. The most commonly prescribed antibiotics were the combination of ampicillin and gentamycin. Ampicillin was changed to Metronidazole in three of the patients due to the drug allergy.

In our study the most frequently occurred clinical manifestation of meningitis was fever, neck rigidity, and irritability. It was found that the presence of comorbidity, the administration of oxygen and use of corticosteroid was predictors of the treatment outcomes of bacterial meningitis in children. Therefore, in patients with these factors, appropriate meningitis treatment should be encouraged and locally applicable treatment guidelines should be prepared to improve patient outcome. In our study, the patients should be managed by taking broad spectrum Cephalosporin (cefotaxime or ceftriaxone) in children over 3 months old and Ampicillin should be added in young infants less than 3 months old to cover *Listeria monocytogenes.* Finally, the meningitis patients should be given corticosteroid and oxygen as treatment and special attention should be given for patients having co-morbidities.

## Data Availability

The datasets used and/or analyzed during the current study are available from the corresponding author on reasonable request.

## Abbreviations

ABM: Acute Bacterial Meningitis
AOR: Adjusted Odds Ratio
BGH: Bedele General Hospital
BM: Bacterial Meningitis
CI: Confidence Interval
CNS: Central Nervous System
COR: Crude Odds Ratio
CSF: Cerebrospinal Fluid
SPSS: Statistical Package for Social Sciences
TB: Tuberculosis
